# Synthesis of N-(6-(4-(Piperazin-1-yl)phenoxy)pyridin-3-yl)benzenesulfonamide Derivatives for the Treatment of Metabolic Syndrome

**DOI:** 10.1155/2013/201580

**Published:** 2013-12-22

**Authors:** Nabajyoti Deka, Swapnil Bajare, Jessy Anthony, Amrutha Nair, Anagha Damre, Dharmeshkumar Patel, Chandrika B-Rao, H. Sivaramakrishnan, Shivaprakash Jagalur Mutt, Chandan Wilankar, Rosalind Marita

**Affiliations:** ^1^Department of Medicinal Chemistry, Piramal Life Sciences Limited, 1 Nirlon Complex, Goregaon East, Mumbai 400 063, India; ^2^Lupin Limited, Mumbai, India; ^3^Department of Pharmacology, Piramal Life Sciences Limited, 1 Nirlon Complex, Goregaon East, Mumbai 400 063, India; ^4^Department of DMPK, Piramal Life Sciences Limited, 1 Nirlon Complex, Goregaon East, Mumbai 400 063, India; ^5^Department of Discovery Informatics, Piramal Life Sciences Limited, 1 Nirlon Complex, Goregaon East, Mumbai 400 063, India; ^6^Institute of Biomedicine, Department of Physiology, Biocenter of Oulu, Faculty of Medicine, Oulu University, P.O. Box 5000, Aapistie 7, 90014 Oulu, Finland; ^7^Department of Biochemistry, Haffkine Institute for Training, Research and Testing, Parel, Mumbai 400 012, India

## Abstract

Metabolic syndrome is a widely prevalent multifactorial disorder associated with an increased risk of cardiovascular disease and type 2 diabetes mellitus. High plasma levels of insulin and glucose due to insulin resistance are a major component of the metabolic disorder. Thiazolidinediones (TZDs) are potent PPAR*γ* ligand and used as insulin sensitizers in the treatment of type 2 diabetes mellitus. They are potent insulin-sensitizing agents but due to adverse effects like hepatotoxicity, a safer alternative of TZDs is highly demanded. Here we report synthesis of N-(6-(4-(piperazin-1-yl)phenoxy)pyridin-3-yl)benzenesulfonamide derivatives as an alternate remedy for insulin resistance.

## 1. Introduction

**Figure 1:**
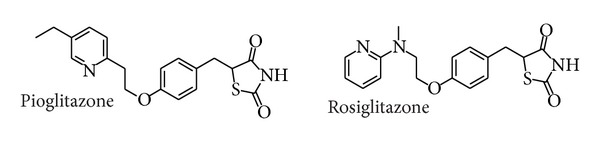
Structure of known PPAR*γ* ligands.

Metabolic disorder is a highly widespread clinical entity. Although obesity and insulin resistance are not synonymous with the metabolic syndrome, they are integral features in this derangement of adipocyte physiology and carbohydrate metabolism. PPARs play a key role in adipocyte differentiation and insulin sensitivity [1]. They are lipid sensors known to govern numerous biological processes. There are three PPAR subtypes (*α*, *β*, and *γ*) and they regulate the expression of numerous genes involved in a variety of metabolic pathways [2]. Roles of PPAR*α* and PPAR*γ* are now quite well known, particularly since their pharmacologic ligands have been marketed. PPAR*α* and PPAR*γ* are the target of the lipid-normalizing class of fibrates (e.g., fenofibrate and gemfibrozil) and the antidiabetic class of thiazolidinediones (e.g., rosiglitazone and pioglitazone), respectively [3]. PPAR*γ* is expressed most abundantly in adipose tissue and is a master regulator of adipogenesis [4, 5]. Thiazolidinediones (Figure 1), selective activators of PPAR*γ*, have been marketed as antidiabetic drugs. They enhance insulin action, improve glycemic control, reduce the level of glycohemoglobin (HbA_1C_), and have variable effects on serum triglyceride levels in type 2 diabetic patients. Despite their efficacy, they possess a number of side effects [6, 7], including weight gain and peripheral edema, increased risks of congestive heart failure, and increased rate of bone fracture. The weight gain is likely due to multiple interacting factors, including increased adiposity and fluid retention. Moreover, the assumption that TZD treatment causes a significant increase in the risk of myocardial infarction and an increase in the risk of death from cardiovascular events in patients with type 2 diabetes. More importantly, TZDs treatment was recently shown to decrease bone formation and accelerated bone loss in healthy and insulin resistant individuals [8]. Such major safety concerns led to development failure of a large number of PPAR agonists.

**Figure 2:**
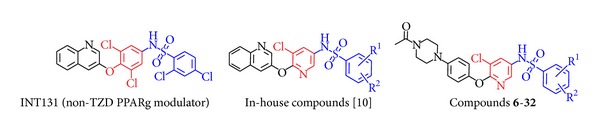
Structure of INT131, in-house compounds [10], and representation of compounds **6–32**.

During the last decade, a major investment was made by the pharmaceutical industry to develop safer PPAR*γ* modulator. This effort led to the description of several unique non-TZD partial PPAR*γ* agonists with a potential for reduced side effects. INT131 (Figure 2), for example, a novel, nonthiazolidinedione (TZD), selective peroxisome proliferator-activated receptor gamma (PPAR*γ*) modulator [9], is in development by InteKrin Therapeutics Inc. for the treatment of type 2 diabetes mellitus. Here we report synthesis of N-(6-(4-(piperazin-1-yl)phenoxy)pyridin-3-yl)benzenesulfonamide derivatives as an alternate remedy for insulin resistance. Compounds were screened for adipogenesis in 3T3-L1 adipocytes and in PPAR*γ* transactivation assay. They were found efficacious in rodent models of T2DM.

## 2. Results and Discussion

### 2.1. Concept

In our ongoing discovery program for the development of non-TZD PPAR*γ* agonist [10], we have synthesized N-(6-(quinolin-3-yloxy)pyridin-3-yl)benzenesulfonamide derivatives. Compounds from that series showed significant activation in PPAR*γ* transactivation assay and moderate-to-high adipogenic activity. The compounds also exhibited plasma glucose reduction in *db/db* mice. Structure activity relationship study of the series revealed that replacement of 3-quinolinoxy with a phenyl ring with piperazine substitution at 4-position reduced PPAR*γ* activation.

Thus we developed a series of novel compounds on the basis of chemical structure of known PPAR*γ* modulator INT131 and our in-house compound [10] with the intent of reducing peroxisome proliferator-activated receptor gamma (PPAR*γ*) related activity and retaining *in vivo* efficacy. Here we report the synthesis of N-(6-(4-(piperazin-1-yl)phenoxy)pyridin-3-yl)benzenesulfonamide derivatives (**6–32**) as a promising novel scaffold for the development of antihyperglycemic agent. The compounds were tested for adipogenic activity in the presence of insulin in 3T3-L1 cells at a concentration of 20 *μ*g/mL. They exhibited adipogenesis enhancement in 3T3-1 adipocytes. The compounds were also tested for agonist activity on PPAR in transiently transfected HEK 293 cells at a concentration of 10 *μ*M. They exhibited weak-to-moderate agonist activity and interestingly they displayed significant improvement in glycemic control in *db/db* mice. They did not show significant increase in body weight and no adverse effect in terms of increase in liver weight or liver enzymes was observed.

### 2.2. Docking

**Figure 3:**
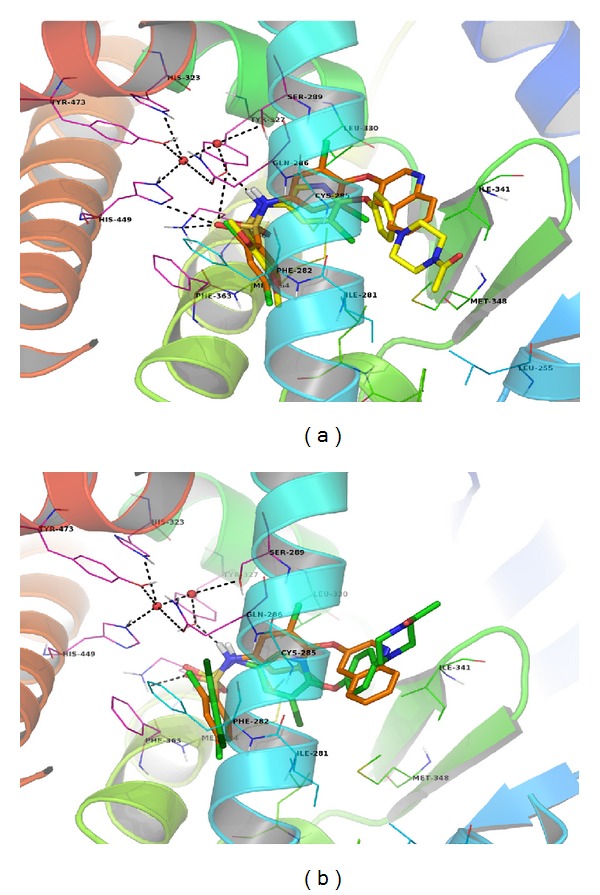
Superimposition of INT131 (brown) and compounds **7** (yellow) and **6** (green) inside the PPAR*γ* active pocket.

Representative compounds **6** and **7** were docked in the active site of PPAR*γ* pocket (PDB ID-3FUR downloaded from Protein Data Bank: http://www.rcsb.org/pdb/) and compared with binding pattern of INT131. They exhibited comparable similarities and interactions in both H3 and AF2 helix. Compounds **6** and **7** superpose very well on INT131 and both the compounds hug the H3 helix with hydrophobic interactions and form water-mediated H-bonds with AF2 helix (Figure 3).

Figure 3 shows that INT131 wraps around the H3 helix without getting close to the AF2 helix. While direct interaction with AF2 helix plays a major role in the binding affinity of PPAR*γ* agonists, INT131 has water-mediated interactions with AF2 helix [11], which may reduce the strength of binding. In case of INT131, dichlorobenzene at the sulfonamide part has dense hydrophobic interactions with hydrophobic cavity of Phe360, Phe363, Ile281, Phe282, Cys285, Leu356. In compound **7** dichlorobenzene is replaced by dimethoxybenzene. While hydrophobic interactions of aromatic ring are maintained as in INT131, interactions of Cl are lost and oxygen atoms have no hydrophobic interactions. But compound **6** has similar hydrophobic interaction due to 2,4-dichlorobenzene as in INT131. Both the compounds **6** and **7 **exhibit H-bond interaction with Gln286, Ser289, His323, His449, and Tyr473 through water molecule and directly with Tyr327and Lys367.

Gold score for INT131 is 90.65 while compound **6 **and** 7** exhibited a comparable GS of 78.09 and 87.26, respectively. Hydrogen bonding score for compound **6** is 6.11 and 7.42 for compounds **7**. These docking data indicated that compounds **6** and **7** exhibited comparable affinity for PPAR*γ* and similar ligand-protein interactions.

### 2.3. Synthesis

**Scheme 1:**
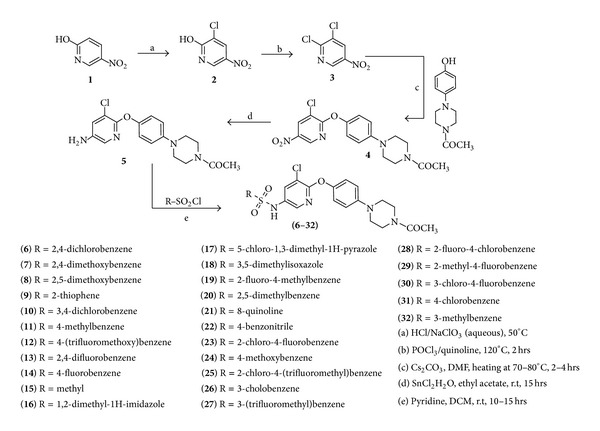
Synthesis of N-(6-(4-(piperazin-1-yl)phenoxy)pyridin-3-yl)benzenesulfonamide derivatives.

The synthesis of novel non-TZD ligands, N-(5-chloro-6-(quinolin-3-yloxy)pyridin-3-yl)benzenesulfonamide derivatives (**6–32**), is depicted in Scheme 1. Synthesis of the target scaffold involved introduction of 4-phenyl piperazine moiety to pyridyl sulfonamide unit. For this purpose, commercially available 2-hydroxy-5-nitropyridine (**1**) was treated with concentrated HCl followed by aqueous NaClO_3_ and the resulting 2-hydroxy-3-chloro-5-nitropyridine (**2**) was converted to 2,3-dichloro-5-nitropyridine (**3**) by using POCl_3_ at 120°C. 1-(4-(4-Hydroxyphenyl)piperazin-1-yl)ethanone and 2,3-dichloro-5-nitropyridine (**3**) were treated with Cs_2_CO_3_ in DMF to get 1-(4-(4-(3-chloro-5-nitropyridin-2-yloxy)phenyl)piperazin-1-yl)ethanone (**4**) which was reduced by SnCl2*·*H_2_O in ethyl acetate to corresponding amine, 1-(4-(4-(5-amino-3-chloropyridin-2-yloxy)phenyl)piperazin-1-yl)ethanone (**5**).

### 2.4. Biological Activity

N-(6-(4-(Piperazin-1-yl)phenoxy)pyridin-3-yl)benzenesulfonamide derivatives were screened in 3T3-L1 cells to evaluate their adipogenic activity. In 3T3-L1 cells, they showed stimulation of adipocyte differentiation (Table 1) indicating that these compounds may activate PPAR*γ* and related target genes involved in adipogenesis. Surprisingly these compounds did not exhibit significant PPAR*γ* activation in transiently transfected HEK 293 cells at a concentration of 10 *μ*M.

In our present work, adipogenesis is used as primary screening followed by PAR*γ* transactivation assay [12, 13]. Adipogenesis is a functional phenotypic based assay involving interplay of multiple targets which implies the possibility of multiple ligand-protein interactions. So inference of a meaningful structure activity relationship may not be precise but it seems that an aromatic sulfonamide is obligatory for adipogenic activity. Thus compound **15** where phenyl ring is replaced by methyl group exhibited very weak *E*_max_ in adipogenesis assay.

Following the identification of compounds with good intrinsic *in vitro* adipogenic activity and weak PPAR*γ* agonism, it was necessary to determine whether these compounds would retain useful *in vivo* efficacy in diabetes models while exhibiting an enhanced safety profile with respect to known PPAR*γ* mechanism-based liabilities. Easily monitored liabilities include body weight gain. *In vivo* testing in rodent models was used to ascertain whether this objective could be attained. A *db/db* mouse, a genetic model of obese, insulin resistant T2DM, was used to evaluate the *in vivo* efficacy of analogs with adequate pharmacokinetic profiles. After oral administration of compound **6** in *db/db* mice at dose of 100 mg/kg, *C*_max_ observed was 207.1 *μ*g/mL at 2.0 h with exposure, AUC_last_, of 1349 h∗*μ*g/mL.

**Figure 4:**
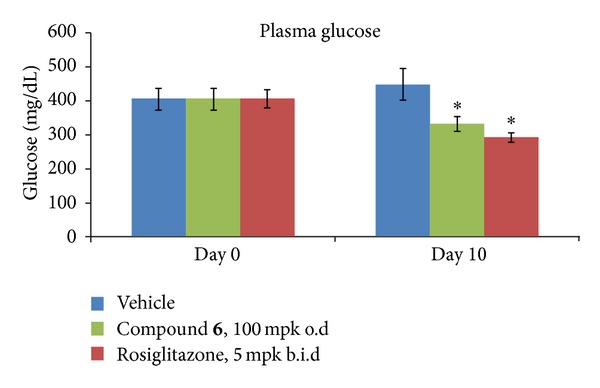
Effect of compound **6** on plasma glucose in *db/db* mice.

Based on the *in vitro* activity and pharmacokinetic profile, a number of compounds were tested in the *db/db* mouse model. Compound **6** showed a better pharmacokinetic profile and its efficacy data are described in detail. Compound **6** effectively reduces hyperglycemia in the *db/db* model (Figure 4) after a treatment of 100 mg/kg/o.d for 10 days (26% plasma glucose reduction). In the same model the known anti diabetic agent, rosiglitazone, was effective as well in its glucose reduction activity (5 mg/kg/b.i.d, 35% glucose reduction).

**Figure 5:**
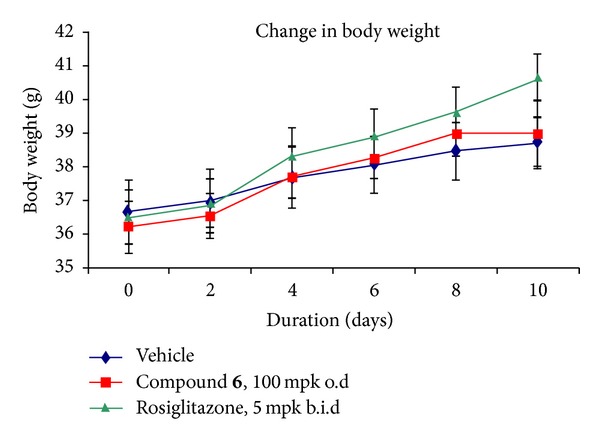
Effect of compound **6** on body weight gain in *db/db* mice.

**Figure 6:**
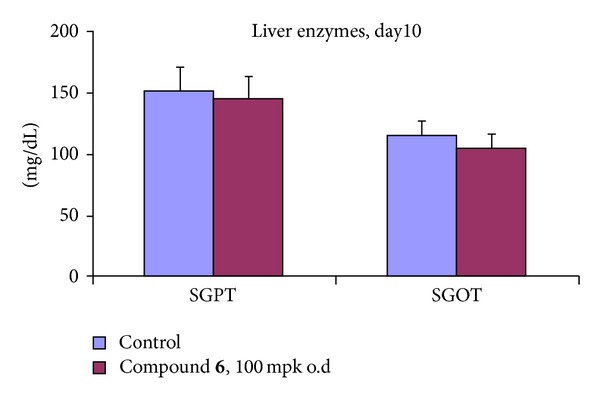
Effect of compound **6** on liver enzymes.

Body weight gain was measured in the *db/db* mouse and only 7.12% of increase was observed with compound **6** even at a higher dose of 100 mg/kg/o.d, while significantly a 10.33% of body weight gain was noted with rosiglitazone at a comparatively lower dose of 5 mg/kg/b.i.d. Therefore, the efficacy study with compound **6** displayed significant glucose lowering activity (Figure 4) and a reduced effect on body weight gain as compared to rosiglitazone (Figure 5) indicating that compound **6**, a non-TZD PPAR*γ* modulator, possesses important pharmacological advantages relative to the TZD PPAR*γ* agonist rosiglitazone in this animal model. No elevation of liver enzymes (Figure 6) and increase in liver weight were observed with compound **6** at the effective dose of 100 mpk.

## 3. Conclusion

Here in this study we report design and synthesis of N-(5-chloro-6-(quinolin-3-yloxy)pyridin-3-yl)benzenesulfonamide derivatives as novel non-TZD PPAR*γ* agonist for the development of a safer antidiabetic agent. The synthesized molecules exhibited adipogenesis activity and PPAR*γ* agonism leading to antidiabetic effect in *db/db* mice. In comparison with the PPAR*γ* full agonist rosiglitazone, adverse effects such as body weight gain were attenuated. Further investigations to enhance these desirable profiles are ongoing.

## 4. Experimental

### 4.1. General Conditions

All reagents and solvents were obtained from Sigma Aldrich and used as received. ^1^H-NMR and ^13^C-NMR spectra were obtained on a “Bruker 300 MHz” instrument equipped with a 5 mm ^1^H/^13^C/X (BBO) probe and the solvent indicated with tetramethylsilane as an internal standard. The data so obtained were processed and analyzed by using Bruker software, XWIN NMR version 3.5. Analytical HPLC was run using a Zorbax Eclipse XDB-C8 3.5 *μ*m 4.6 × 75 mm column eluting with a mixture of acetonitrile and water containing 0.1% trifluoroacetic acid with a 5 minute gradient of 10%–100%. MS results were obtained on “ESI-QTOF” instruments of Bruker Daltonics (model MicrotofQ). Ten *μ*L of each sample (fraction) was injected. The sample was ionized using Electron Spray Ionisation technique and analyzed using quadruple time of flight. The mobile phase used was acetonitrile and of 0.1% formic acid (50 : 50) with a flow rate of 0.2 mL/min. The samples were analyzed both in the positive mode and negative mode by direct injection mode. Liquid chromatography/mass spectroscopy studies have been carried out using “Agilent 1100 Series/esquire 4000” instrument of Bruker Daltonics. The same analytical HPLC method was used with “Phenomenex Luna C18” column. 0.1% of formic acid and acetonitrile (80 : 20) were used as mobile phase. Ten *μ*L of sample was injected on ESI source. Ion source parameters were employed as required. Nebulizer pressure = 45 psi, dry gas = 12 Lit/min, dry temp = 350°C, scan = 50 to 2200, capillary voltage = 4500, polarity was checked for both positive and negative modes. The data so obtained were processed and analyzed by using HyStar software.

Automated column chromatography was performed on a CombiFlash Rf 200 (Teledyne Isco Inc.).

### 4.2. Docking Protocol

The protein structure of PPAR*γ* (PDB ID-3FUR), which was used for docking, was downloaded from Protein Data Bank (http://www.rcsb.org/pdb/). This is a cocrystal structure of PPAR*γ* with INT131. Compounds were docked to the binding site by means of CCDC's GOLD (Genetic Optimization for Ligand Docking) software, version 5.1. The binding region for the docking study was defined as all atoms within 6 Å radius sphere centered on the centroid of the INT131. Thirty genetic algorithm (GA) runs were performed with automatic settings for each compound. The scoring function, GoldScore, implemented in GOLD was used to rank the docking positions of compound.

### 4.3. *In Vitro* Assays

The compounds were tested for adipogenic activity in the presence of insulin in 3T3-L1 cells at a concentration of 20 *μ*g/mL [12]. The adipogenic activity in the presence of potent PPAR*γ* full agonist, rosiglitazone, at 1 *μ*M was defined as 100%, and the maximum adipogenic activity in the presence of the test compound was defined as *E*_max_ (%). All values have been generated with *n* = 2. The compounds were tested for agonist activity on PPAR in transiently transfected HEK 293 cells at a concentration of 10 *μ*M [13]. The transcriptional activity in the presence of potent PPAR*γ* full agonist (1 *μ*M) was defined as 100%, and the maximum transcriptional activity in the presence of the test compound was defined as *E*_max_ (%). Cells were procured from American Type Culture Collection (ATCC).

### 4.4. PK Study

Pharmacokinetic parameters were assessed following oral dosing (100 mg/kg) using a suspension formulation (using 0.5% CMC and Tween 80; dosing volume: 10 mL/kg). Female *db/db* mice were weighed and the compounds were administered orally (*n* = 4 per time point). Blood samples were withdrawn at 0.08, 0.25, 0.5, 0.75, 1.0, 2.0, 6.0, and 8.0 h after dosing. Plasma samples were maintained on ice before being centrifuged (4°C for 5 min at 1411 g), and aliquots were stored at −80°C pending the assay. Concentrations of the compounds were determined using an HPLC method developed at Piramal Healthcare Limited. Pharmacokinetic parameters were determined by noncompartmental analysis using WinNonlin Professional (version 4.1). *C*_max_ and *T*_max_ were taken directly from the plasma concentration-time profile. The area under the curve from time 0 to the last blood sampling time (AUC_0–*t*_) was calculated using the linear trapezoidal rule. The area under the curve extrapolated to infinity (AUC_inf_) was calculated by using the plasma concentration at time *t* divided by slope *λz*, where *λz* is estimated by linear regression of the terminal log-linear phase of the plasma concentration-time curve. Terminal plasma elimination half life (*T*_1/2_) was calculated as 0.693/*λz*.

*HPLC Method for PK Study*. Plasma samples were thawed on the day of analysis at room temperature. For processing, an aliquot (100 *μ*L) of each plasma sample was precipitated by vortex mixing with 1.0 mL of acetonitrile for 5 minutes. The samples were then centrifuged (10000 rpm, 5 min) at 4°C. Supernatants (850 *μ*L) were transferred to glass tubes and evaporated to dryness under nitrogen (15 psi, 37°C) for 20 minutes. The dried residues were reconstituted in 100 *μ*L mixture of acetonitrile-methanol (1 : 1% v/v). The reconstituted samples were vortexed for 1 minute and centrifuged, and the resulting supernatants were subjected to HPLC analysis.

The chromatographic system consisted of a Thermo Finnigan Surveyor LC pump with a Photodiode Array Detector (Thermo Electron Corporation, San Jose, USA). Compound **6** was separated at 25°C on a Thermo BDS Hypersil C18 column of 250 × 4.6 mm I.D. and particle size of 5 *μ*m. The mobile phase composed of two solvents: Solvent A, 100% HPLC grade acetonitrile, and Solvent B, 0.01 M ammonium acetate containing 0.5% v/v triethylamine, pH adjusted to 5 with glacial acetic acid. The mobile phase was run at a flow rate of 1 mL/min using the following gradient program (Time/%A): 0/0, 10/100, 15/100, 15.01/0, and 22/0. Absorbance was measured at 254 nm. Unknown concentrations of compound **6** in the plasma samples were determined using a calibration curve in mouse plasma at concentrations ranging from 0.025 to 25 *μ*g/mL. Plasma samples exceeding the upper limit of quantification were diluted with control mouse plasma before precipitation. A linear relationship for compound **6** (*r* ^2^ = 0.999) was obtained when peak areas of linearity samples were plotted against concentration. Coefficients of variation were lower than 10%, whereas accuracy ranged from 90% to 115%. Quality control samples were found to be within normal acceptance criteria for bioanalytical methods.

### 4.5. *In Vivo* Experiment

All animal experiments were performed according to procedures approved by the CPCSEA and as per the IAEC guidelines. In brief, from 5-to-7-week-old male *db/db* mice, bred at Piramal Enterprises Limitedy, were fed a chow diet. The mice were treated with the respective compounds, and body weight and biochemical parameters were evaluated at the end of the study.

### 4.6. Analytical Data

*1-(4-(4-((3-Chloro-5-nitropyridin-2-yl)oxy)phenyl)piperazin-1-yl)ethanone* (**4**). In a 250 mL of round bottom flask, 22.0 g (0.1 mol) of 1-(4-(4-hydroxyphenyl)piperazin-1-yl)ethanone was placed and 100 mL of dry dimethyl formamide was added. To the stirred solution, 39.0 g (0.12 mol) of Cs_2_CO_3_ was added at 0°C. Stirring was continued and after 30 minutes 1.9 g (0.1 mol) of 2,3-dichloro-5-nitro pyridine (**3**) was added. Stirring was continued further for 3-4 hours and the reaction was monitored by TLC. After completion, the reaction mixture was poured into ice-water and extracted with ethyl acetate. Organic layer was separated and dried over sodium sulfate (Na_2_SO_4_). Solvent was removed under vacuum and to the resulting mass was added water (50 mL), extracted with ethyl acetate, dried over sodium sulfate, and concentrated under vacuum to obtain crude 1-(4-(4-((3-chloro-5-nitropyridin-2-yl)oxy)phenyl)piperazin-1-yl)ethanone that was purified using flash chromatography (Yield 78%).

^1^H NMR (300 MHz, DMSO-d6): *δ* 9.21 (d, *J* = 2.4 Hz, 2H), 8.98 (d, *J* = 2.4 Hz, 1H), 7.04 (d, *J* = 8.1 Hz, 2H), 6.99 (d, *J* = 8.1 Hz, 2H), 3.57 (m, 4H), 3.17 (m, 2H), 3.08 (m, 2H), 2.06 (s, 3H).

HRMS (*m*/*z*): [M]^+^ calculated for C_17_H_17_ClN_4_O_4_, 376.0938; found, 376.0947.

*1-(4-(4-((5-Amino-3-chloropyridin-2-yl)oxy)phenyl)piperazin-1-yl)ethanone* (**5**). In a 250 mL of round bottom flask, 9.4 g (0.025 mol) of 1-(4-(4-((3-chloro-5-nitropyridin-2-yl)oxy)phenyl)piperazin-1-yl)ethanone was placed and 100 mL of ethyl acetate was added. To the stirred solution 220 g (0.10 mol) of SnCl_2_ *·*2H_2_O was added at room temperature and stirring was continued for 8 hours. After completion (monitored by TLC), the solvent evaporated and 25 mL of water added followed by 1 N NaOH solution (pH was maintained between 9 and 10). Ethyl acetate (50 mL × 3) was used for extraction. The combined ethyl acetate layers were dried over sodium sulfate and concentrated under reduced pressure. The crude compound obtained was purified by column chromatography using 30% ethyl acetate in petroleum ether solvent system which gave 6.0 g (0.0175 mol) of 1-(4-(4-((5-amino-3-chloropyridin-2-yl)oxy)phenyl)piperazin-1-yl)ethanone. Yield 70%.

^1^H NMR (300 MHz, DMSO-d6): *δ* 7.91 (d, *J* = 2.4 Hz, 1H), 7.16 (d, *J* = 2.4 Hz, 1H), 7.02 (d, *J* = 7.5 Hz, 2H), 6.98 (d, *J* = 7.5 Hz, 2H), 5.82 (s, 2H), 3.55 (m, 4H), 3.14 (m, 2H), 3.05 (m, 2H), 2.03 (s, 2H).

HRMS (*m*/*z*): [M]^+^ calculated for C_17_H_19_ClN_4_O_2_, 346.1197; found, 346.1213.

*General Procedure for the Synthesis of Compounds* (**6–32**). In a 100 mL round bottom flask, 867 mg (2.5 mmol) of the amine, 1-(4-(4-((5-amino-3-chloropyridin-2-yl)oxy)phenyl)piperazin-1-yl)ethanone, was placed and dissolved with dry DCM and 2 mL of pyridine was added. To the stirred solution, 2.5 mmol of desired sulfonyl chloride was added. The reaction mixture was stirred at room temperature for 5-6 hours and monitored by TLC. After completion, the solvent evaporated and the resulting crude product was purified using ethyl acetate and pet ether solvent system in automated column chromatography (CombiFlash Rf 200 from Teledyne Isco Inc.). All purified compounds (**6–32**) were characterized by spectral analysis.

*N-(6-(4-(4-Acetylpiperazin-1-yl)phenoxy)-5-chloropyridin-3-yl)-2,4-dichlorobenzenesulfonamide (* ***6***). Yield 72%.

^1^H NMR (300 MHz, DMSO-d6): *δ* 10.91 (s, 1H), 7.99 (d, *J* = 8.4 Hz, 1H), 7.86 (d, *J* = 2.1 Hz, 1H), 7.72 (d, *J* = 2.4 Hz, 1H), 7.66 (d, *J* = 2.4 Hz, 1H), 7.59 (dd, *J* = 2.4, 8.4 Hz, 1H), 6.96 (m, 4H), 3.56 (m, 4H), 3.11 (m, 2H), 3.03 (m, 2H), 2.01 (s, 3H). ^13^C NMR (300 MHz, DMSO-d6): *δ* 169.8, 152.7, 150.9, 145.8, 141.7, 139.8, 138.9, 133.7 (2C), 131.8 (2C), 128.3, 122.8, 121.2 (2C), 116.4 (2C), 114.3, 54.2 (2C), 45.9 (2C), 22.1.

HRMS (*m*/*z*): [M]^+^ calculated for C_23_H_21_Cl_3_N_4_O_4_S, 554.0349; found, 554.0351.

*N-(6-(4-(4-Acetylpiperazin-1-yl)phenoxy)-5-chloropyridin-3-yl)-3,4-dimethoxybenzenesulfonamide *(**7**). Yield 74%.

^1^H NMR (300 MHz, DMSO-d6): *δ* 10.26 (s, 1H), 7.64 (s, 2H), 7.28 (d, *J* = 8.4 Hz, 1H), 7.25 (s, 1H), 7.07 (d, *J* = 8.4 Hz, 1H), 6.94 (m, 4H), 3.78 (s, 3H), 3.74 (s, 3H), 3.55 (m, 4H), 3.09 (m, 2H), 3.02 (m, 2H), 2.06 (s, 3H). ^13^C NMR (300 MHz, DMSO-d6): *δ* 169.8, 154.1, 152.7, 150.8, 145.7, 141.6, 134.1, 133.6, 122.8, 121.2 (2C), 119.4 (2C), 116.7, 115.1 (2C), 114.3, 113.2, 57.2 (2C), 54.2 (2C), 45.9 (2C), 22.1.

HRMS (*m*/*z*): [M]^+^ calculated for C_25_H_27_ClN_4_O_6_S, 546.1340; found, 546.1335.

*N-(6-(4-(4-Acetylpiperazin-1-yl)phenoxy)-5-chloropyridin-3-yl)-2,5-dimethoxybenzenesulfonamide* (**8**). Yield 73%.

^1^H NMR (300 MHz, DMSO-d6): *δ* 10.69 (s, 1H), 7.91 (d, *J* = 2.1 Hz, 1H), 7.85 (d, *J* = 2.4 Hz, 1H), 7.56 (d, *J* = 7.8 Hz, 1H), 7.18 (dd, *J* = 2.4, 7.8 Hz, 1H), 7.12 (d, *J* = 2.1 Hz, 1H), 6.98 (m, 4H), 4.12 (s, 6H), 3.55 (m, 4H), 3.11 (m, 2H), 3.04 (m, 2H), 2.02 (s, 3H). ^13^C NMR (300 MHz, DMSO-d6): *δ* 169.8, 152.7, 151.4, 150.1, 149.8, 145.7, 141.5, 133.6, 122.8, 121.2 (2C), 120.6, 119.4, 116.7, 115.1 (2C), 114.3, 56.2 (2C), 54.2 (2C), 45.9 (2C), 22.1.

HRMS (*m*/*z*): [M]^+^ calculated for C_25_H_27_ClN_4_O_6_S, 546.1340; found, 546.1335.

*N-(6-(4-(4-Acetylpiperazin-1-yl)phenoxy)-5-chloropyridin-3-yl)thiophene-2-sulfonamide* (**9**). Yield 67%.

^1^H NMR (300 MHz, DMSO-d6): *δ* 10.58 (s, 1H), 7.96 (m, 1H), 7.72 (d, *J* = 2.1 Hz, 1H), 7.66 (d, *J* = 2.4 Hz, 1H), 7.55 (m, 1H), 7.15 (t, *J* = 4.5 Hz, 1H), 6.97 (m, 4H), 3.56 (m, 4H), 3.11 (m, 2H), 3.04 (m, 2H), 2.03 (s, 3H). ^13^C NMR (300 MHz, DMSO-d6): *δ* 169.8, 152.7, 150.1, 145.7, 141.5, 133.6, 128.3 (2C), 127.1, 126.9, 122.8, 121.2 (2C), 115.1 (2C), 114.3, 54.2 (2C), 45.9 (2C), 22.1.

HRMS (*m*/*z*): [M]^+^ calculated for C_21_H_21_ClN_4_O_4_S_2_, 492.0693; found, 492.0689.

*N-(6-(4-(4-Acetylpiperazin-1-yl)phenoxy)-5-chloropyridin-3-yl)-3,4dichlorobenzenesulfonamide* (**10**). Yield 71%.

^1^H NMR (300 MHz, DMSO-d6): *δ* 10.61 (s, 1H), 7.92 (d, *J* = 1.8 Hz, 1H), 7.86 (d, *J* = 8.4 Hz, 1H), 7.66 (m, 3H), 6.96 (m, 4H), 3.32 (m, 4H), 3.11 (m, 2H), 3.04 (m, 2H), 2.02 (s, 3H). ^13^C NMR (300 MHz, DMSO-d6): *δ* 169.8, 152.7, 150.1, 145.7, 141.5, 140.3, 137.7, 134.8, 133.6, 131.5, 129.0, 127.9, 122.8, 121.2 (2C), 115.1 (2C), 114.3, 54.2 (2C), 45.9 (2C), 22.1.

HRMS (*m*/*z*): [M]^+^ calculated for C_23_H_21_Cl_3_N_4_O_4_S, 554.0349; found, 554.0345.

*N-(6-(4-(4-Acetylpiperazin-1-yl)phenoxy)-5-chloropyridin-3-yl)-4-methoxybenzenesulfonamide* (**11**). Yield 69%.

^1^H NMR (300 MHz, DMSO-d6): *δ* 10.48 (s, 1H), 7.63 (d, *J* = 2.4 Hz, 1H), 7.61 (d, *J* = 2.4 Hz, 1H), 7.58 (d, *J* = 8.1 Hz, 2H), 7.37 (d, *J* = 8.1 Hz, 2H), 6.94 (m, 4H), 3.36 (m, 4H), 3.09 (m, 2H), 3.02 (m, 2H), 2.33 (s, 3H), 1.97 (s, 3H). ^13^C NMR (300 MHz, DMSO-d6): *δ* 169.8, 152.7, 150.1, 145.7, 141.5, 138.6, 137.7, 133.6, 130.5, 129.4 (2C), 122.8, 121.2 (2C), 115.1 (2C), 114.3, 54.2 (2C), 45.9 (2C), 22.1.

HRMS (*m*/*z*): [M]^+^ calculated for C_24_H_25_ClN_4_O_5_S, 516.1234; found, 516.1242.

*N-(6-(4-(4-Acetylpiperazin-1-yl)phenoxy)-5-chloropyridin-3-yl)-4-methylbenzenesulfonamide* (**12**). Yield 72%.

^1^H NMR (300 MHz, DMSO-d6): *δ* 10.58 (s, 1H), 7.86 (d, *J* = 1.5 Hz, 1H), 7.83 (d, *J* = 1.5 Hz, 1H), 7.67 (dd, *J* = 2.1, 7.8 Hz, 2H), 7.58 (dd, *J* = 2.1, 7.8 Hz, 2H), 6.95 (m, 4H), 3.55 (m, 4H), 3.09 (m, 2H), 3.02 (m, 2H), 2.02 (s, 3H). ^13^C NMR (300 MHz, DMSO-d6): *δ* 169.8, 152.7, 150.1, 145.7, 144.1, 141.5, 135.3, 133.6, 129.5 (2C), 128.2 (2C), 125.3, 122.8, 121.2 (2C), 115.1 (2C), 114.3, 54.2 (2C), 45.9 (2C), 22.1.

HRMS (*m*/*z*): [M]^+^ calculated for C_24_H_25_ClN_4_O_4_S, 500.1285; found, 500.1312.

*N-(6-(4-(4-Acetylpiperazin-1-yl)phenoxy)-5-chloropyridin-3-yl)-2,4-difluorobenzenesulfonamide* (**13**). Yield 68%.

^1^H NMR (300 MHz, DMSO-d6): *δ* 10.86 (s, 1H), 7.90 (m, 1H), 7.71 (d, *J* = 2.4 Hz, 1H), 7.68 (d, *J* = 2.4 Hz, 1H), 7.55 (m, 1H), 7.25 (m, 1H), 6.95 (m, 4H), 3.55 (m, 4H), 3.09 (m, 2H), 3.03 (m, 2H), 2.08 (s, 3H). ^13^C NMR (300 MHz, DMSO-d6): *δ* 169.8, 165.4, 160.9, 152.7, 150.1, 145.7, 141.5, 133.6, 131.5, 122.8 (2C), 121.2 (2C), 115.1 (2C), 114.3, 112.5, 106.1, 54.2 (2C), 45.9 (2C), 22.1.

HRMS (*m*/*z*): [M]^+^ calculated for C_23_H_21_ClF_2_N_4_O_4_S, 522.0940; found, 522.0949.

*N-(6-(4-(4-Acetylpiperazin-1-yl)phenoxy)-5-chloropyridin-3-yl)-4-fluorobenzenesulfonamide* (**14**). Yield 68%.

^1^H NMR (300 MHz, DMSO-d6): *δ* 10.48 (s, 1H), 7.80 (m, 2H), 7.64 (d, *J* = 2.1 Hz, 1H), 7.61 (d, *J* = 2.1 Hz, 1H), 7.40 (m, 2H), 6.96 (m, 4H), 3.55 (m, 4H), 3.09 (m, 2H), 3.03 (m, 2H), 2.02 (s, 3H). ^13^C NMR (300 MHz, DMSO-d6): *δ* 169.8, 167.2, 152.7, 150.1, 145.7, 141.5, 136.4, 133.6, 131.8 (2C), 122.8, 121.2 (2C), 116.9 (2C), 115.1 (2C), 114.3, 54.2 (2C), 45.9 (2C), 22.1.

HRMS (*m*/*z*): [M]^+^ calculated for C_23_H_22_ClFN_4_O_4_S, 504.1034; found, 504.1041.

*N-(6-(4-(4-Acetylpiperazin-1-yl)phenoxy)-5-chloropyridin-3-yl)methanesulfonamide* (**15**). Yield 65%.

^1^H NMR (300 MHz, DMSO-d6): *δ* 9.89 (s, 1H), 7.89 (d, *J* = 2.1 Hz, 1H), 7.82 (d, *J* = 2.1 Hz, 1H), 7.01 (m, 4H), 3.59 (m, 4H), 3.14 (m, 2H), 3.05 (m, 2H), 2.04 (s, 3H). ^13^C NMR (300 MHz, DMSO-d6): *δ* 169.8, 152.7, 150.1, 145.7, 141.5, 133.6, 122.8, 121.2 (2C), 115.1 (2C), 114.3, 54.2 (2C), 45.9 (2C), 43.1, 22.1.

HRMS (*m*/*z*): [M]^+^ calculated for C_18_H_21_ClN_4_O4_6_S, 424.0972; found, 424.0979.

*N-(6-(4-(4-Acetylpiperazin-1-yl)phenoxy)-5-chloropyridin-3-yl)-1,2-dimethyl-1H-imidazole-4-sulfonamide* (**16**). Yield 63%.

^1^H NMR (300 MHz, DMSO-d6): *δ* 10.36 (s, 1H), 7.78 (s, 1H), 7.77 (d, *J* = 2.4 Hz, 1H), 7.74 (d, *J* = 2.4 Hz, 1H), 6.98 (m, 4H), 3.65 (s, 3H), 3.56 (m, 4H), 3.11 (m, 2H), 3.05 (m, 2H), 2.27 (s, 3H), 2.04(s, 3H). ^13^C NMR (300 MHz, DMSO-d6): *δ* 169.8, 152.7, 150.1, 145.7, 144.1, 141.5, 133.6, 126.6, 123.5, 122.8, 121.2 (2C), 115.1 (2C), 114.3, 54.2 (2C), 45.9 (2C), 34.5, 22.1, 14.7.

HRMS (*m*/*z*): [M]^+^ calculated for C_22_H_25_ClN_6_O_4_S, 504.1347; found, 504.1353.

*N-(6-(4-(4-Acetylpiperazin-1-yl)phenoxy)-5-chloropyridin-3-yl)-5-chloro-1,3-dimethyl-1H-pyrazole-4-sulfonamide* (**17**). Yield 65%.

^1^H NMR (300 MHz, DMSO-d6): *δ* 10.54 (s, 1H), 7.71 (m, 2H), 6.98 (m, 4H), 3.73 (s, 3H), 3.57 (m, 4H), 3.12 (m, 2H), 3.05 (m, 2H), 2.22 (s, 3H), 2.04 (s, 3H). ^13^C NMR (300 MHz, DMSO-d6): *δ* 169.8, 152.7, 151.9, 150.1, 145.7, 141.5, 134.6, 133.6, 122.8, 122.1, 121.2 (2C), 115.1 (2C), 114.3, 54.2 (2C), 45.9 (2C), 38.3, 22.1, 13.2.

HRMS (*m*/*z*): [M]^+^ calculated for C_22_H_24_Cl_2_N_6_O_4_S, 538.0957; found, 538.0963.

*N-(6-(4-(4-Acetylpiperazin-1-yl)phenoxy)-5-chloropyridin-3-yl)-3,5-dimethylisoxazole-4-sulfonamide* (**18**). Yield 63%.

^1^H NMR (300 MHz, DMSO-d6): *δ* 10.64 (s, 1H), 7.75 (d, *J* = 2.1 Hz, 2H), 6.99 (m, 4H), 3.58 (m, 4H), 3.13 (m, 2H), 3.06 (m, 2H), 2.44 (s, 3H), 2.34 (s, 3H), 2.04 (s, 3H). ^13^C NMR (300 MHz, DMSO-d6): *δ* 169.8, 159.1, 153.7, 151.3, 150.1, 145.7, 141.5, 133.6, 122.8, 122.1, 121.2 (2C), 115.1 (2C), 114.3, 101.5, 54.2 (2C), 45.9 (2C), 22.1, 12.2, 9.8.

HRMS (*m*/*z*): [M]^+^ calculated for C_22_H_24_ClN_5_O_5_S, 505.1187; found, 505.1198.

*N-(6-(4-(4-Acetylpiperazin-1-yl)phenoxy)-5-chloropyridin-3-yl)-2-fluoro-4-methylbenzenesulfonamide* (**19**). Yield 68%.

^1^H NMR (300 MHz, DMSO-d6): *δ* 10.49 (s, 1H), 7.65 (s, 2H), 7.46 (m, 3H), 6.98 (m, 4H), 4.02 (m, 4H), 3.10 (m, 2H), 3.05 (m, 2H), 2.48 (s, 3H), 2.01 (s, 3H). ^13^C NMR (300 MHz, DMSO-d6): *δ* 169.8, 159.1, 152.7, 150.1, 145.7, 142.8, 141.5, 133.6, 129.8, 125.1, 124.5, 122.8, 121.2 (2C), 117.3, 115.1 (2C), 114.3, 54.2 (2C), 45.9 (2C), 22.4, 21.2.

HRMS (*m*/*z*): [M]^+^ calculated for C_24_H_24_ClN_4_O_4_S, 518.1191; found, 518.1202.

*N-(6-(4-(4-Acetylpiperazin-1-yl)phenoxy)-5-chloropyridin-3-yl)-2,5-dimethylbenzenesulfonamide* (**20**). Yield 70%.

^1^H NMR (300 MHz, DMSO-d6): *δ* 10.36 (s, 1H), 7.65 (d, *J* = 2.1 Hz, 1H), 7.62 (d, *J* = 2.1 Hz, 1H), 7.33 (m, 2H), 7.27 (d, *J* = 2.4 Hz, 1H), 6.98 (m, 4H), 3.57 (m, 4H), 3.15 (m, 2H), 3.08 (m, 2H), 2.48 (s, 6H), 2.02 (s, 3H). ^13^C NMR (300 MHz, DMSO-d6): *δ* 169.8, 152.7, 150.1, 145.7, 141.5, 139.9, 136.8, 134.6, 133.5, 132.3, 130.1, 127.6, 122.1, 121.2 (2C), 115.1 (2C), 114.3, 54.2 (2C), 45.9 (2C), 23.1, 22.3, 21.1.

HRMS (*m*/*z*): [M]^+^ calculated for C_25_H_27_ClN_4_O_6_S, 546.1340; found, 545.1351.

*N-(6-(4-(4-Acetylpiperazin-1-yl)phenoxy)-5-chloropyridin-3-yl)quinoline-8-sulfonamide* (**21**). Yield 71%.

^1^H NMR (300 MHz, DMSO-d6): *δ* 10.57 (s, 1H), 9.13 (dd, *J* = 2.4, 8.4 Hz, 1H), 8.79 (dd, *J* = 2.4, 8.4 Hz, 1H), 8.68 (dd, *J* = 2.1, 7.8 Hz, 1H), 8.46 (dd, *J* = 2.1, 7.8 Hz, 1H), 8.18 (t, *J* = 7.8 Hz, 1H), 7.99 (t, *J* = 8.4 Hz, 1H), 7.91 (d, *J* = 2.1 Hz, 1H), 7.14 (d, *J* = 2.1 Hz, 1H), 6.98 (m, 4H), 3.55 (m, 4H), 3.12 (m, 2H), 3.03 (m, 2H), 2.02 (s, 3H). ^13^C NMR (300 MHz, DMSO-d6): *δ* 169.8, 152.9, 151.8, 149.9 (2C), 145.6, 141.5, 138.1, 137.4, 133.5, 130.1, 129.6, 125.3, 124.1, 122.7, 121.5, 120.9 (2C), 115.1 (2C), 114.3, 54.2 (2C), 45.9 (2C), 21.1.

HRMS (*m*/*z*): [M]^+^ calculated for C_26_H_24_ClN_5_O_4_S, 537.1238; found, 537.1242.

*N-(6-(4-(4-Acetylpiperazin-1-yl)phenoxy)-5-chloropyridin-3-yl)-4-cyanobenzenesulfonamide* (**22**). Yield 69%.

^1^H NMR (300 MHz, DMSO-d6): *δ* 10.73 (s, 1H), 8.06 (d, *J* = 8.1 Hz, 2H), 7.89 (d, *J* = 7.8 Hz, 2H), 7.67 (d, *J* = 2.1 Hz, 2H), 6.97 (m, 4H), 3.56 (m, 4H), 3.12 (m, 2H), 3.05 (m, 2H), 2.02 (s, 3H). ^13^C NMR (300 MHz, DMSO-d6): *δ* 169.8, 152.7, 150.1, 145.7, 141.5, 133.5, 132.9 (2C), 127.6 (2C), 122.1, 121.2 (2C), 119.6, 116.8, 115.1 (2C), 114.3, 54.2 (2C), 45.9 (2C), 21.1.

HRMS (*m*/*z*): [M]^+^ calculated for C_24_H_22_ClN_5_O_4_S, 511.1081; found, 511.1089.

*N-(6-(4-(4-Acetylpiperazin-1-yl)phenoxy)-5-chloropyridin-3-yl)-2-chloro-4-fluorobenzenesulfonamide* (**23**). Yield 70%.

^1^H NMR (300 MHz, DMSO-d6): *δ* 10.86 (s, 1H), 8.06 (dd, *J* = 6.0, 8.7 Hz, 1H), 7.72 (m, 2H), 7.66 (d, *J* = 2.1 Hz, 1H), 7.38 (m, 1H), 6.95 (m, 4H), 3.55 (m, 4H), 3.10 (m, 2H), 3.05 (m, 2H), 2.02 (s, 3H). ^13^C NMR (300 MHz, DMSO-d6): *δ* 169.8, 168.5, 152.7, 150.1, 145.7, 141.5, 136.4, 134.2, 133.5, 131.3, 122.1, 121.2 (2C), 119.2, 115.1 (2C), 114.3, 113.2, 54.2 (2C), 45.9 (2C), 21.2.

HRMS (*m*/*z*): [M]^+^ calculated for C_23_H_21_Cl_2_FN_4_O_4_S, 538.0645; found, 538.0653.

*N-(6-(4-(4-Acetylpiperazin-1-yl)phenoxy)-5-chloropyridin-3-yl)-4-methoxybenzenesulfonamide* (**24**). Yield 71%.

^1^H NMR (300 MHz, DMSO-d6): *δ* 10.30 (s, 1H), 7.66 (m, 2H), 7.63 (m, 2H), 7.08 (m, 2H), 6.94 (m, 4H), 3.79 (s, 3H), 3.55 (m, 4H), 3.09 (m, 2H), 3.02 (m, 2H), 2.02 (s, 3H). ^13^C NMR (300 MHz, DMSO-d6): *δ* 169.8, 164.9, 152.7, 150.1, 145.7, 141.5, 133.5, 132.3, 127.6 (2C), 122.1, 121.2 (2C), 115.1 (2C), 114.6 (2C), 113.2, 59.8, 54.2 (2C), 45.9, 21.1.

HRMS (*m*/*z*): [M]^+^ calculated for C_24_H_25_ClN_4_O_4_S, 516.1234; found, 516.1242.

*N-(6-(4-(4-Acetylpiperazin-1-yl)phenoxy)-5-chloropyridin-3-yl)-2-chloro-4-(trifluoromethyl)benzenesulfonamide* (**25**). Yield 68%.

^1^H NMR (300 MHz, DMSO-d6): *δ* 11.08 (s, 1H), 8.20 (d, *J* = 8.1 Hz, 1H), 8.13 (d, *J* = 2.1 Hz, 1H), 7.90 (dd, *J* = 2.1, 8.1 Hz, 1H), 7.73 (d, *J* = 2.4 Hz, 1H), 7.69 (d, *J* = 2.4 Hz, 1H), 6.93 (m, 4H), 3.54 (m, 4H), 3.09 (m, 2H), 3.02 (m, 2H), 2.01 (s, 3H). ^13^C NMR (300 MHz, DMSO-d6): *δ* 169.8, 152.7, 150.1, 145.7, 144.1, 141.5, 140.3, 133.5, 132.3, 130.1, 126.3 (2C), 124.6, 122.1, 121.2 (2C), 115.1 (2C), 114.3, 54.2 (2C), 45.9 (2C), 21.2.

HRMS (*m*/*z*): [M]^+^ calculated for C_24_H_21_Cl_2_F_3_N_4_O_4_S, 588.0613; found, 588.0620.

*N-(6-(4-(4-Acetylpiperazin-1-yl)phenoxy)-5-chloropyridin-3-yl)-3-chlorobenzenesulfonamide* (**26**). Yield 70%.

^1^H NMR (300 MHz, DMSO-d6): *δ* 10.56 (s, 1H), 7.74 (m, 2H), 7.57 (m, 4H), 6.96 (m, 4H), 3.56 (m, 4H), 3.11 (m, 2H), 3.01 (m, 2H), 2.02 (s, 3H). ^13^C NMR (300 MHz, DMSO-d6): *δ* 169.8, 152.7, 150.1, 145.7, 142.2, 141.5, 135.7, 133.5, 132.3, 131.5, 127.6, 126.4, 122.1, 121.2 (2C), 115.1 (2C), 114.3, 54.2 (2C), 45.9 (2C), 21.2.

HRMS (*m*/*z*): [M]^+^ calculated for C_23_H_22_Cl_2_N_4_O_4_S, 520.0739; found, 520.0744.

*N-(6-(4-(4-Acetylpiperazin-1-yl)phenoxy)-5-chloropyridin-3-yl)-3-(trifluoromethyl)benzenesulfonamide* (**27**). Yield 67%.

^1^H NMR (300 MHz, DMSO-d6): *δ* 10.59 (s, 1H), 8.06 (d, *J* = 7.8 Hz, 1H), 7.98 (m, 2H), 7.82 (t, *J* = 7.8 Hz, 1H), 7.64 (m, 2H), 6.98 (m, 4H), 3.55 (m, 4H), 3.10 (m, 2H), 3.05 (m, 2H), 2.02 (s, 3H). ^13^C NMR (300 MHz, DMSO-d6): *δ* 169.8, 152.7, 150.1, 145.7, 141.5, 140.8, 139.9, 133.5, 132.3, 131.6, 130.3, 129.4, 125.2, 123.9, 122.1, 121.2 (2C), 115.1 (2C), 114.3, 54.2 (2C), 45.9 (2C), 21.2.

HRMS (*m*/*z*): [M]^+^ calculated for C_24_H_22_ClF_3_N_4_O_4_S, 554.1002; found, 554.1013.

*N-(6-(4-(4-Acetylpiperazin-1-yl)phenoxy)-5-chloropyridin-3-yl)-4-chloro-2-fluorobenzenesulfonamide* (**28**). Yield 69%.

^1^H NMR (300 MHz, DMSO-d6): *δ* 10.89 (s, 1H), 7.83 (d, *J* = 7.8 Hz, 1H), 7.77 (m, 1H), 7.73 (d, *J* = 2.4 Hz, 1H), 7.70 (d, *J* = 2.4 Hz, 1H), 7.47 (dd, *J* = 1.5, 8.7 Hz, 1H), 6.96 (m, 4H), 3.56 (m, 4H), 3.11 (m, 2H), 3.06 (m, 2H), 2.02 (s, 3H). ^13^C NMR (300 MHz, DMSO-d6): *δ* 169.8, 159.8, 152.7, 150.1, 145.7, 141.5, 139.8, 133.5, 131.3, 125.6, 124.7, 122.1, 121.2 (2C), 119.2, 115.1 (2C), 114.3, 54.2 (2C), 45.9 (2C), 21.1.

HRMS (*m*/*z*): [M]^+^ calculated for C_23_H_21_Cl_2_FN_4_O_4_S, 538.0645; found, 538.0621.

*N-(6-(4-(4-Acetylpiperazin-1-yl)phenoxy)-5-chloropyridin-3-yl)-4-fluoro-2-methylbenzenesulfonamide* (**29**). Yield 69%.

^1^H NMR (300 MHz, DMSO-d6): *δ* 10.61 (s, 1H), 7.89 (m, 1H), 7.69 (d, *J* = 2.1 Hz, 1H), 7.62 (d, *J* = 2.1 Hz, 1H), 7.31 (m, 1H), 7.22 (m, 1H), 6.95 (m, 4H), 3.56 (m, 4H), 3.10 (m, 2H), 3.04 (m, 2H), 2.58 (s, 3H), 2.03 (s, 3H). ^13^C NMR (300 MHz, DMSO-d6): *δ* 169.8, 167.1, 152.7, 150.1, 145.7, 141.5, 139.3, 135.5, 133.5, 128.7, 122.1, 121.2 (2C), 117.5, 115.1 (2C), 114.3, 113.7, 54.2 (2C), 45.9 (2C), 22.9, 21.2.

HRMS (*m*/*z*): [M]^+^ calculated for C_24_H_24_Cl_3_FN_4_O_4_S, 518.1191; found, 518.1212.

*N-(6-(4-(4-Acetylpiperazin-1-yl)phenoxy)-5-chloropyridin-3-yl)-3-chloro-4-fluorobenzenesulfonamide* (**30**). Yield 70%.

^1^H NMR (300 MHz, DMSO-d6): *δ* 10.56 (s, 1H), 7.94 (m, 1H), 7.65 (m, 4H), 6.97 (m, 4H), 3.56 (m, 4H), 3.11 (m, 2H), 3.05 (m, 2H), 2.03 (s, 3H). ^13^C NMR (300 MHz, DMSO-d6): *δ* 169.8, 163.1, 152.7, 150.1, 145.7, 141.5, 137.8, 133.5, 129.9, 122.1, 121.8, 121.1 (2C), 118.3, 115.1 (2C), 114.3, 54.2 (2C), 46.1 (2C), 21.2.

HRMS (*m*/*z*): [M]^+^ calculated for C_23_H_21_Cl_2_FN_4_O_4_S, 538.0645; found, 538.0653.

*N-(6-(4-(4-Acetylpiperazin-1-yl)phenoxy)-5-chloropyridin-3-yl)-4-chlorobenzenesulfonamide* (**31**). Yield 70%.

^1^H NMR (300 MHz, DMSO-d6): *δ* 10.54 (s, 1H), 7.73 (m, 2H), 7.66 (m, 4H), 6.96 (m, 4H), 3.56 (m, 4H), 3.12 (m, 2H), 3.06 (m, 2H), 2.02 (s, 3H). ^13^C NMR (300 MHz, DMSO-d6): *δ* 169.8, 152.7, 150.1, 145.7, 141.5, 138.9, 138.6, 133.6, 130.2 (2C), 129.6 (2C), 122.1, 121.2 (2C), 115.1 (2C), 114.3, 54.2 (2C), 46.1 (2C), 21.2.

HRMS (*m*/*z*): [M]^+^ calculated for C_23_H_22_Cl_2_N_4_O_4_S, 520.0739; found, 520.0746.

*N-(6-(4-(4-Acetylpiperazin-1-yl)phenoxy)-5-chloropyridin-3-yl)-3-methylbenzenesulfonamide* (**32**). Yield 68%.

^1^H NMR (300 MHz, DMSO-d6): *δ* 10.41 (s, 1H), 7.66 (d, *J* = 2.4 Hz, 1H), 7.63 (d, *J* = 2.1 Hz, 1H), 7.55 (m, 1H), 7.51 (m, 1H), 7.46 (m, 2H), 6.96 (m, 4H), 3.56 (m, 4H), 3.13 (m, 2H), 3.06 (m, 2H), 2.34 (s, 3H), 2.01 (s, 3H). ^13^C NMR (300 MHz, DMSO-d6): *δ* 169.8, 152.7, 150.1, 145.7, 141.5, 40.1, 139.7, 133.5, 132.3, 129.9, 127.6, 125.3, 122.1, 121.2 (2C), 115.1 (2C), 114.3, 54.1 (2C), 45.9 (2C), 22.1, 21.2.

HRMS (*m*/*z*): [M]^+^ calculated for C_24_H_25_ClN_4_O_4_S, 500.1285; found, 500.1293.

## Figures and Tables

**Table 1:** Compounds with *in vitro* biological activity.

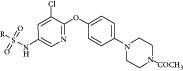			
Compound number	R	Adipogenesis $E_{\max}$ (%)^a^	PPAR*γ* agonism $E_{\max}$ (%)^b^

**6**	2,4-Dichlorobenzene	90	36.4
**7**	3,4-Dimethoxybenzene	67	9.12
**8**	2,5-Dimethoxybenzene	72	18.5
**9**	2-Thiophene	69	11.3
**10**	3,4-Dichlorobenzene	75	19.2
**11**	4-Methylbenzene	73	17.8
**12**	4-(Trifluoromethoxy)benzene	89	26.8
**13**	2,4-Difluorobenzene	81	25.7
**14**	4-Fluorobenzene	74	9.4
**15**	Methyl	52	6.5
**16**	1,2-Dimethyl-1H-imidazole	69	8.2
**17**	5-Chloro-1,3-dimethyl-1H-pyrazole	69	8.5
**18**	3,5-Dimethylisoxazole	69	8.5
**19**	2-Fluoro-4-methylbenzene	78	14.6
**20**	2,5-Dimethylbenzene	79	22.5
**21**	8-Quinoline	82	29.3
**22**	4-Benzonitrile	85	27.6
**23**	2-Chloro-4-fluorobenzene	85	27.5
**24**	4-Methoxybenzene	82	25.0
**25**	2-Chloro-4-(trifluoromethyl)benzene	87	31.7
**26**	3-Cholobenzene	71	11.2
**27**	3-(Trifluoromethyl)benzene	76	19.1
**28**	2-Fluoro-4-chlorobenzene	75	20.9
**29**	2-Methyl-4-fluorobenzene	65	8.17
**30**	3-Chloro-4-fluorobenzene	72	16.8
**31**	4-Chlorobenzene	78	19.3
**32**	3-Methylbenzene	62	11.4

All values have been generated with a *n* = 2.

^a^The compounds were tested for adipogenic activity in the presence of insulin in 3T3-L1 cells at a concentration of 20 *µ*g/mL [12]. The adipogenic activity in the presence of potent PPAR*γ* full agonist, rosiglitazone, at 1 *µ*M was defined as 100%, and the maximum adipogenic activity in the presence of the test compound was defined as *E*_max_ (%).

^b^The compounds were tested for agonist activity on PPAR in transiently transfected HEK 293 cells at a concentration of 10 *µ*M [13]. The transcriptional activity in the presence of rosiglitazone (1 *µ*M) was defined as 100%; the maximum transcriptional activity in the presence of the test compound was defined as *E*_max_ (%).
